# Johnston’s Manual of Chemistry

**Published:** 1843-11

**Authors:** 


### JOHNSTON'S MANUAL OF CHEMISTRY.

This work is intended for a text-book in Colleges and other Seminaries of learning, and as a manual for such learners it is sufficient recommendation, that it contains for its basis Turner's Elements of Chemistry, such parts of that admirable work being omitted as would be out of place in a text-book. It is disencumbered of many details of facts, and discussions of opinions and theories, which are rather calculated to confuse the young student, than to interest him, at the same time that it is fuller than any of the manuals designed for the same class of learners. With all the clearness of Turner, it is more practical, going more into the application of chemistry to the arts, which, with its diminished size, renders it a capital book for schools, into which, it is hoped, it will be extensively introduced.

